# Emergency department referrals for CT imaging of extremity soft tissue infection: before and during the COVID-19 pandemic

**DOI:** 10.1093/bjro/tzae016

**Published:** 2024-08-22

**Authors:** Andrew Nanapragasam, Lawrence M White

**Affiliations:** Department of Medical Imaging, University of Toronto, Toronto, ON M5G 2C4, Canada; Department of Medical Imaging, University of Toronto, Toronto, ON M5G 2C4, Canada

**Keywords:** soft tissue infection, cellulitis, phlegmon, abscess, ulcer, necrotising fasciitis, COVID-19

## Abstract

**Objectives:**

To evaluate the incidence and spectrum of findings in patients referred for CT imaging of extremity soft tissue infection in the adult emergency department (ED) setting before and during the COVID-19 pandemic.

**Methods:**

Two hundred thirteen CT exams in the pre-COVID cohort (February 1, 2018-January 31, 2020) and 383 CT exams in the COVID cohort (February 1, 2020-January 31, 2022) were evaluated in this multicentre, retrospective study. Demographic information and clinical histories were collected, along with regional data on COVID-19 hospitalizations and deaths.

**Results:**

Comparable age and sex distribution was found in the pre-COVID (average age of 53.5 years; male: female ratio of 71:29) and COVID (average age of 54.6 years; male: female ratio of 69:31) cohorts. The frequency of reported clinical risk factors (diabetes mellitus, injected drug use, prior surgery, animal bite) was not significantly different between the two cohorts. Findings of simultaneous involvement of both superficial and deep soft tissue infection on CT imaging were significantly higher in the COVID cohort (53.4%) than in the pre-COVID cohort (33.7%). CT findings of phlegmon (49.1% vs 22.1%), ulcers (48.8% vs 30%), osteomyelitis (21.7% vs 13.1%), as well as localized (18.8% vs 11.7%) and extensive (3.7% vs 2.3%) soft tissue gas were significantly more common in the COVID cohort than in the pre-COVID cohort.

**Conclusions:**

During the COVID-19 pandemic, the number of ED CT exams for the evaluation of extremity soft tissue infection increased, with this imaging also showing more advanced disease. Pandemic-related modifications to human behaviour and re-distribution of healthcare resources may underlie these observed changes.

**Advances in knowledge:**

This multi-centre study shows an increase in extremity soft tissue infection presenting to the ED during the pandemic. This finding is important for future pandemic preparations, as it can aid in the decision-making process around resource allocation.

## Introduction

The World Health Organization (WHO) declared COVID-19 to be a “Public Health Emergency of International Concern” on January 30, 2020, and they subsequently upgraded the disease classification to a “pandemic” on March 11, 2020.^1^^,^^2^ The first confirmed COVID-19 case in Canada was recorded in Toronto on January 25, 2020.^3^ The morbidity and mortality directly related to the COVID-19 virus are well-documented, with data relating to infections and deaths available for most countries globally. However, there are various secondary healthcare consequences related to the pandemic, the full effect of which we are yet to quantify.

Changes in human behaviour and healthcare resource allocation in response to the pandemic are thought to have impacted the patterns of disease presentation and delivery of care. A possible example of this is severe soft tissue infection. Soft tissue infections are typically treated in the outpatient setting, assuming they are detected and accurately diagnosed early on in the disease process. With the reduction in outpatient appointments and a potential reluctance to attend outpatient medical services due to apprehension about COVID-19 exposure, there is a risk that soft tissue infections may present later and with more severe changes. Soft tissue infection encompasses a spectrum of disease from superficial soft tissue cellulitis to life-threatening necrotizing fasciitis, which may need emergency department (ED) attendance and cross-sectional imaging evaluation. This study sought to evaluate and compare the use of CT imaging in ED patients referred for assessment of suspected extremity soft tissue infection, during and immediately preceding the COVID-19 pandemic.

## Methods

Institutional research ethics board (REB) approval with waived consent was obtained for this retrospective study. All CT exams of the extremities ordered through the ED of 3 large urban academic medical centres were identified through the departmental radiology information system for the 2-year timeframe immediately preceding the COVID-19 pandemic (February 1, 2018-January 31, 2020) and a 2-year time period during the first through forth waves of the pandemic (February 1, 2020-January 31, 2022), where healthcare services were disrupted in response to the virus spread.

The provided clinical history and dictated reports of ED extremity CT exams were searched for any of the following keyword terms: “infection”, “ulcer”, “cellulitis”, “collection”, “abscess”, “phlegmon”, “gas”, “necrotising”, “necrotizing”, “fasciitis”, “nec fasc”, in order to identify exams performed for the evaluation of possible soft tissue infection. Each of the CT reports and clinical histories were reviewed in consensus by 2 subspecialty trained musculoskeletal radiology “readers” (AN, LMW) with 6 and 30 years of radiology experience, respectively, to identify CT exams not related to the evaluation of suspected soft tissue infection (eg, trauma, vascular disease), and these studies were excluded from the data set.

**Figure 1.**
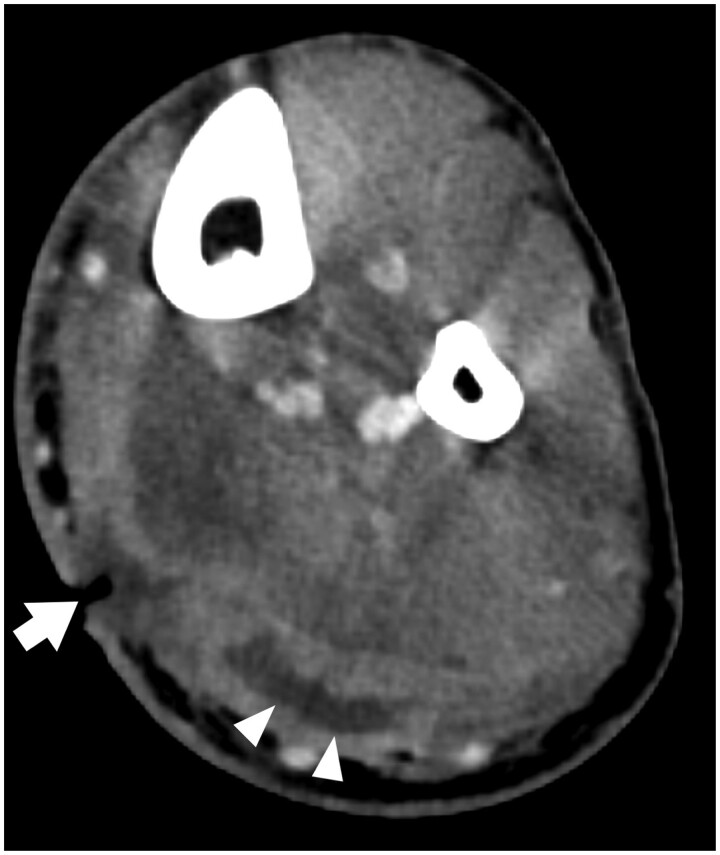
Axial venous phase contrast enhanced CT of the calf showing a cutaneous ulcer (arrow) with a low attenuation fluid collection (arrowheads) in the underlying posterior muscle compartment.

**Figure 2.**
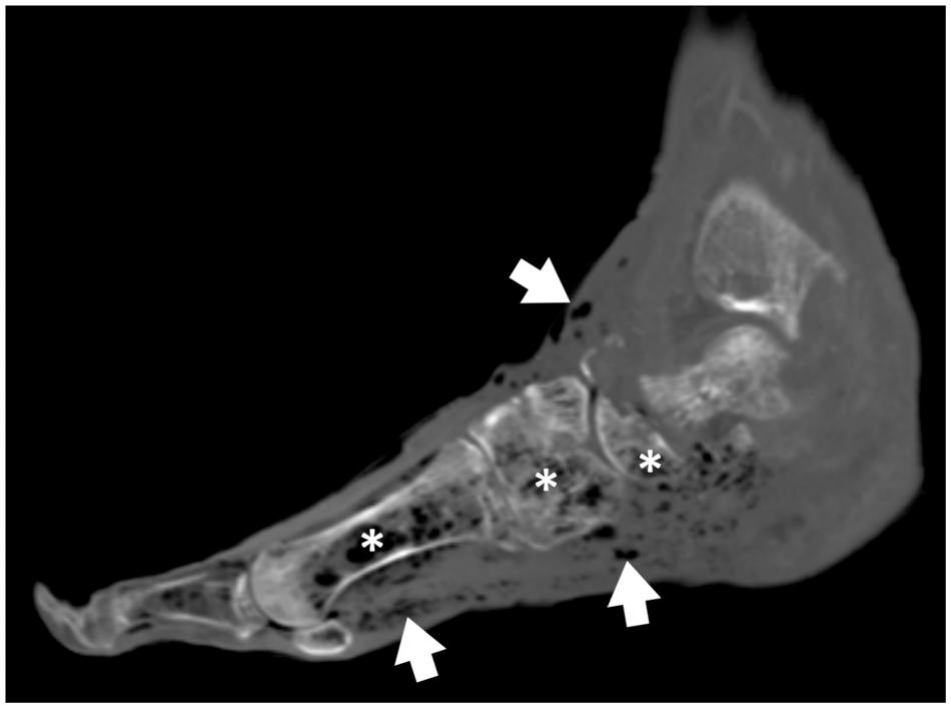
Sagittal venous phase contrast enhanced CT of the foot showing extensive soft tissue gas locules (arrows) and intra-osseous gas indicating osteomyelitis (asterisks).

For each of the remaining CT exams, patient age, sex, provided clinical history, and background patient clinical details including documented history of diabetes mellitus, persons who inject drugs (PWID), local surgical intervention, or animal bite wound, were recorded. Each CT exam was additionally categorized by anatomic region(s) of infective change (shoulder, arm, forearm, hand/wrist, hip, thigh, leg, ankle/foot). Data on whether the exam was performed with or without the administration of intravenous contrast material were also collected. All CT exams were additionally reviewed by both readers in-consensus in determination of the presence or absence of each of the following: superficial or deep compartmental changes, cellulitis, phlegmon, ulceration, fluid collection/abscess, localized or diffuse soft tissue gas, and osteomyelitis. Infectious processes considered “superficial” were those with changes limited to the dermis/subcutaneous fat, and while these changes may contact the underlying superficial fascia, they do not penetrate through the fascia into the muscle compartment. The presence of ill-defined reticular oedematous change in the subcutaneous fat on CT was used as the diagnostic feature of cellulitis.^4^ Phlegmon was defined as an area of markedly thickened soft tissue, showing diffuse enhancement on contrast-enhanced imaging, with no areas of loculated low-attenuation fluid to suggest abscess formation.^5^ Ulceration was defined as an irregular defect in the contour of the skin, often covered by dressing/bandage material on CT imaging (Figure 1).^5^ A focal soft tissue swelling or mass with thick or irregular walls and heterogeneous central low-attenuation fluid was used as diagnostic criteria for an extremity soft tissue collection/abscess (Figure 1).^6^ Soft tissue gas, which can be seen focally at sites of an injury or more diffusely in severe necrotizing infections with gas-forming organisms, was defined as localized (covering an area ≤5 cm) or diffuse (covering an area >5 cm) in nature (Figure 2).^7^ CT features used to define the presence of acute osteomyelitis included findings of osseous erosion, periosteal reaction, and intra-osseous gas (Figure 2).^8^

The compiled data on the number of CT exams of the extremities performed for the investigation of suspected soft tissue infection during the “pre-COVID” (February 1, 2018-January 31, 2020) and “COVID” (February 1, 2020-January 31, 2022) were compared with the overall total number of ED patient visits over the corresponding time-frames. Temporal epidemiologic data regarding waves of COVID-19-related hospitalizations and deaths in the local area were collected, for comparative purposes.^9^

Two-tailed *t*-tests and chi-squared analyses were used to identify potential significant differences.

## Results

Database keyword search identified a total of 291 ED extremity CT exams for the assessment of extremity soft tissue infection between February 1, 2018 and January 31, 2020. Report and clinical history review of each of the 291 exams identified 78 exams which were performed for reasons other than evaluation of soft tissue infection (69 for trauma and 9 vascular studies). These exams were excluded from further analysis. The resulting 213 extremity CT exams performed in ED patients for assessment of suspected soft tissue infection comprised our “pre-COVID” study cohort. The 213 extremity CT exams were performed in 209 different patients. Four patients had repeat imaging at a second ED visit, separated by time intervals of 5, 12, 72, and 84 days. A total of 367 817 ED patient visits were recorded during the pre-COVID time-frame (February 1, 2018-January 31, 2020).

Database keyword search identified a total of 586 ED extremity CT exams for the assessment of extremity soft tissue infection in the time-period between February 1, 2020 and January 31, 2022. Report and clinical history review of each of the 586 exams identified 203 exams which were performed for reasons other than evaluation of soft tissue infection (166 for trauma, 30 vascular studies, 4 for tumour assessment, 2 for avascular necrosis, and 1 aborted exam). These exams were excluded from further analysis. Of the remaining 383 CT exams, 25 patients had repeat imaging at a separate ED visit. Twenty-one patients had 2 visits, and 4 patients had 3 visits. The 383 extremity CT exams performed in 354 different ED patients for evaluation of soft tissue infection during this time period comprised our “COVID” study cohort. A total of 288 190 ED patient visits were recorded over this time-period.

A summary of patient demographics, background clinical history, and CT findings across the study cohort groups is compiled in Table 1. Data on the breakdown of anatomical sites of involvement are available in Supplementary Table S1.

**Table 1.** Statistical comparison of patients presenting for CT imaging of extremity soft tissue infection before and during the COVID-19 pandemic.

		Pre-COVID cohort	COVID cohort	*P*-value
		(Feb 2018-Jan 2020)	(Feb 2020-Jan 2022)	
**Demographic information**	CT exams [*n*] (patients)	213 (209)	383 (354)	
	Mean age [years] (SD)	53.5 (17.7)	54.6 (16.8)	.42 [two-tailed *t*-test]
	Sex distribution	Male: 71%; female: 29%	Male: 69%; female: 31%	.54 [χ^2^(1, *N* = 596) = 0.38]
**Clinical history**	Persons who inject drugs [*n*] (% of cohort)	39 (18.3)	58 (15.1)	.32 [χ^2^(1, *N* = 596) = 1.01]
	Diabetes mellitus [*n*] (% of cohort)	44 (20.7)	89 (23.2)	.47 [χ^2^(1, *N* = 596) = 0.53]
	Prior surgery at the site of infection [*n*] (% of cohort)	21 (9.9)	56 (14.6)	.10 [χ^2^(1, *N* = 596) = 2.76]
	History of an animal bite at the site of infection [*n*] (% of cohort)	4 (1.9)	9 (2.3)	.71 [χ^2^(1, N = 596) = 0.14]
**CT contrast**	Use of intravenous contrast with CT scan [*n*] (% of cohort)	147 (69.0)	243 (63.4)	.17 [χ^2^(1, *N* = 596) = 1.88]
**CT signs of infection**	CT findings positive for an infection [*n*] (% of cohort)	205 (96.2)	367 (95.8)	.33 [χ^2^(1, *N* = 596) = 0.95]
**Extremity involvement on CT**	Upper versus lower extremity involvement [*n*] (% of positive scans)	Upper extremity: 49 (23.9) Lower extremity: 156 (76.1)	Upper extremity: 78 (21.3) Lower extremity: 289 (78.7)	.46 [χ^2^(1, *N* = 572) = 0.53]
**Depth of infection on CT scan**	Superficial changes only [*n*] (% of positive scans)	132 (64.4)	163 (44.4)	**<.001** **[χ^2^(2, *N* = 572) = 8.21]**
	Deep changes only [*n*] (% of positive scans)	4 (2.0)	8 (2.2)	
	Superficial and deep changes [*n*] (% of positive scans)	69 (33.7)	196 (53.4)	
**Infective features on CT imaging**	Cellulitis [*n*] (% of cohort)	192 (90.1)	345 (90.1)	.98 [χ^2^(1, *N* = 596) = 0.0006]
	Phlegmon [*n*] (% of cohort)	47 (22.1)	188 (49.1)	**<.001** **[χ^2^(1, *N* = 596) = 42.8]**
	Ulcer [*n*] (% of cohort)	64 (30.0)	187 (48.8)	**<.001** **[χ^2^(1, *N* = 596) = 19.8]**
	Collection or abscess [*n*] (% of cohort)	65 (30.5)	124 (32.4)	.64 [χ^2^(1, *N* = 596) = 0.22]
	Soft tissue gas: localized [*n*] (% of cohort)	25 (11.7)	72 (18.8)	**.047** **[χ^2^(2, *N* = 596) = 6.12]**
	Soft tissue gas: extensive [*n*] (% of cohort)	5 (2.3)	14 (3.7)	
	Osteomyelitis [*n*] (% of cohort)	28 (13.1)	83 (21.7)	**.01** **[χ^2^(1, *N* = 596) = 6.56]**

Bold indicates statistical significance.

**Figure 3.**
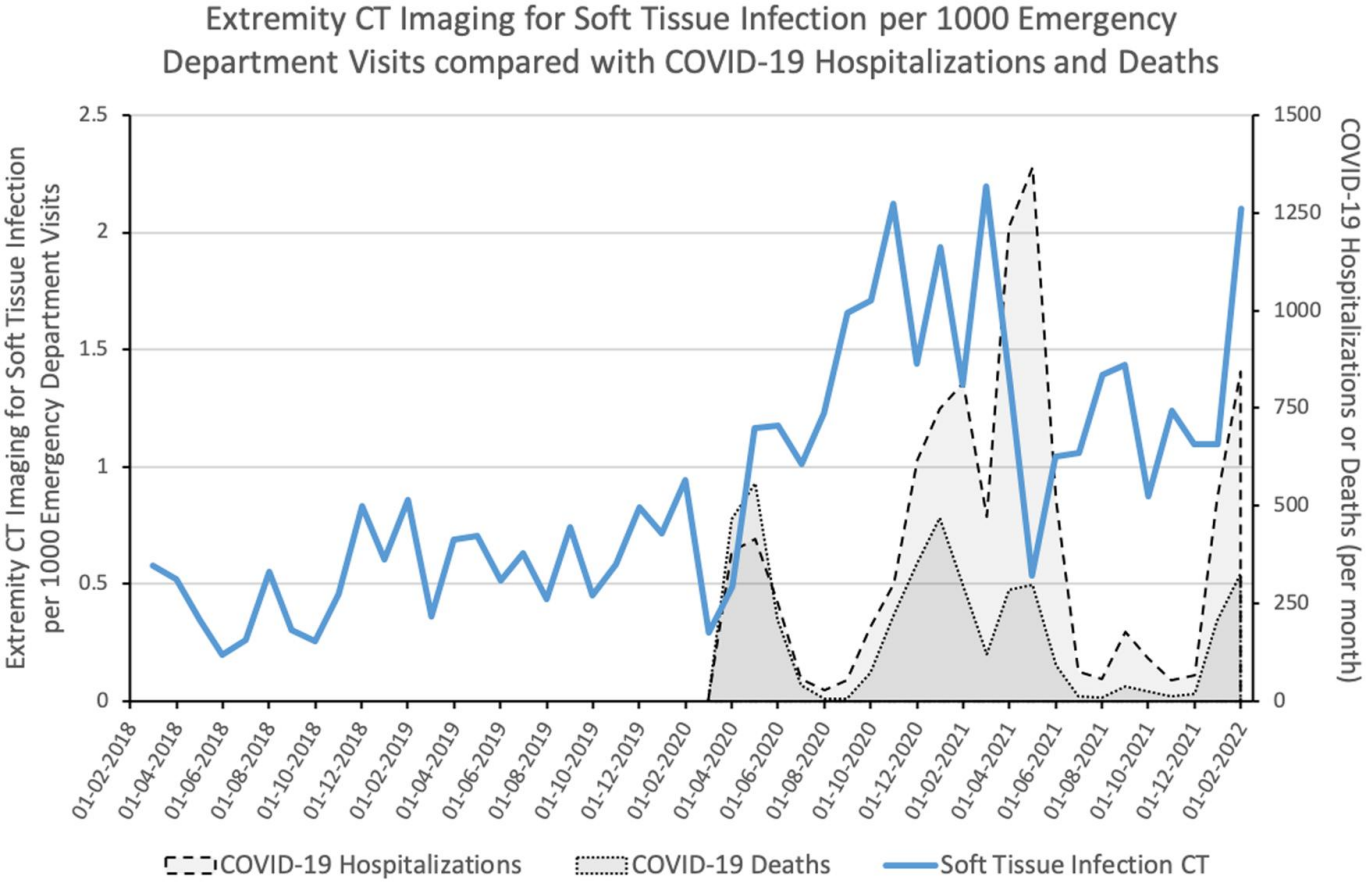
Graph illustrating extremity soft tissue infection CT imaging in the emergency department over time, expressed as a proportion of the total emergency department visits. City-wide data on COVID-19 hospitalizations and deaths have also been included for comparison.

The incidence of ED extremity CT imaging, as a proportion of the total ED visits, is represented graphically over time in Figure 3. Publicly available data documenting regional COVID-19 hospitalizations and deaths has also been included on the same figure for comparative purposes.

The majority of patients referred for CT evaluation of suspected extremity soft tissue infection in the pre-COVID and COVID cohorts were male (71% in the pre-COVID cohort and 69% in the COVID cohort), and no significant difference in the sex or age distribution of patients between the cohorts was shown (Table 1).

The most common clinical background information provided in the CT request for both the pre-COVID and COVID cohorts (Table 1) was a history of a diabetes mellitus, closely followed by a history of injected drug use. Other, less frequent, clinical histories included prior surgery at the site of concern and a history of an animal bite. There were no significant differences observed in the incidence of these clinical factors between the two cohorts.

In addition to a greater number of ED CT exams performed for the evaluation of suspected extremity soft tissue infection in the COVID cohort, there was a significant increase in the proportion of CT exams demonstrating findings of simultaneous involvement of both the superficial and deep soft tissue compartments, and significant increases in the occurrence of phlegmon, ulcers, soft tissue gas, and osteomyelitis observed in the COVID versus pre COVID cohorts (Table 1).

## Discussion

This study has assessed CT imaging of suspected extremity soft tissue infection in the ED setting prior to and during the COVID-19 pandemic. We observed an increase in the absolute number and percentage of ED patients referred for CT imaging of suspected extremity soft tissue infection in the 2 years since the beginning of the pandemic, when compared with the 2-year period immediately prior. Furthermore, the CT exams performed for extremity soft tissue infection during the pandemic show features of more advanced disease, as evidenced by an increase in the proportion of CT exams demonstrating simultaneous involvement of both the superficial and deep compartments of the extremities, and by increases in the incidence of phlegmon, ulcers, soft tissue gas, and osteomyelitis. Findings of our study parallel observations of small-cohort studies that have showed an increased incidence and severity of necrotizing fasciitis during the COVID-19 pandemic.^10^^,^^11^ Another study showed an increased morbidity of upper extremity soft tissue infections in PWID during the pandemic.^12^

The described changes are likely to have a multifactorial aetiology. Changes in the delivery of healthcare during the COVID-19 pandemic are thought to be a contributing factor, with some primary care and outpatient services being moved into the virtual setting, potentially posing access issues for patients. The pandemic also necessitated a temporary change in the focus of healthcare delivery, with personnel and resources being directed to the acute management of COVID-19 infections. This potentially left fewer resources for disease prevention and the management of minor ailments, especially in the primary care setting. In addition to changes in healthcare delivery, there may also have been a change in patients’ behaviour. The importance of social distancing was emphasized to the public, as a means of limiting the spread of infection. This may have resulted in patients being reluctant to seek medical attention. Whether directly related to changes in primary care delivery and/or from fewer patients seeking medical care, an overall decrease in the number of physician-patient interactions was observed in Canada^13–15^ during the pandemic, as well as a 19% decrease in number of antibiotic prescriptions for skin and soft tissue infection provided by family physicians in [location redacted for blinding] (6.4-5.2 prescriptions per 1000 inhabitants) in the initial 8 months of the pandemic compared with the pre-pandemic period.^16^ As a result of these changes, patients with early skin and soft tissue infection may have deteriorated in the community, and only sought care in the ED with more advanced infections of the extremities. Analogous delays in diagnosis and treatment have been documented in cancer care in both paediatric and adult populations during the pandemic.^17–19^

The changes in presentation of soft tissue infection during the pandemic may be explained in part by factors impacting patients with predisposing risk factors to infection (eg, diabetes mellitus). Given potential difficulties accessing primary health services, it is possible that conditions like diabetes were not detected or were poorly controlled, or that patients with skin wounds had limited access to preventative wound care, with the secondary effect of an increase in soft tissue infection.^20^ However, our study did not find a significant difference in the frequency of risk factors (eg, diabetes, PWID) between the pre-COVID and COVID cohorts.

Direct soft tissue abnormalities of the extremities related to COVID-19 have been described including, thrombotic events, soft tissue bleeding secondary to virus-induced coagulopathy, iatrogenic soft tissue changes related to treatment in hospitalized COVID-19 patients,^21^ as well as reports of chilblains-like acral lesions.^22^ However, COVID-19 has not been described as a direct cause of CT soft tissue findings mimicking those of cellulitis or necrotizing soft tissue infection.

The relative contribution of factors to the observed changes in ED patient referrals for CT evaluation of suspected soft tissue infection of the extremities is unclear. However, this study demonstrates evidence of an increase in referrals (absolute number and as percentage of total ED patients) for CT assessment of suspected soft tissue infection, and an increase in the severity of infection in ED patients referred for CT exams, findings of potential relevance to healthcare delivery and planning in future pandemic scenarios.

### Study limitations

There are limitations to the study which should be acknowledged. This study is limited by its retrospective nature; however, the multi-centre patient population of this study is much greater than other similar studies of soft tissue infection during the COVID-19 pandemic.^10^^,^^11^ This study reviewed findings from 3 academic hospitals in a large North American city, and caution should be taken when generalizing the findings to different populations and healthcare delivery environments.

While some soft tissue infections are diagnosed based on the history and physical exam, imaging is commonly used in the evaluation or exclusion of more complicated infections, with CT being the optimal test in the ED setting as a result of its widespread availability, speed, and sensitivity in detection of soft tissue gas and foreign bodies.^23^^,^^24^ The inclusion criteria used in defining our patient study cohorts exclude patients who do not meet the clinical threshold for referral for CT imaging. We acknowledge that this is an indirect index of disease severity and it is likely to vary amongst practitioners. However, clinical recommendations for the use of CT imaging in cases of suspected soft tissue infection, and access to dedicated ED CT imaging across the 3 study hospitals did not change during the study period. Study patients were identified using a keyword search of the departmental radiology information system, and this may have omitted cases. The search parameters were kept broad to minimize this risk, and all CT exam reports and referral histories were reviewed prior to inclusion in the study. No clinical or imaging follow up data was evaluated in the context of the REB approved study protocol, as such, we are unaware of the treatment outcome of patients in this study.

No significant difference in risk factors (diabetes, PWID, prior surgery, animal bite) was found between the two cohorts. However, it should be noted that only risk factors stated in the imaging requisition provided by the referring ED physician were evaluated in the study and as such, predisposing factors may be underestimated due to potentially incomplete clinical histories.

Finally, we have not attempted to perform a financial impact analysis of the changes observed in ED referrals for extremity CT imaging of soft tissue infection. This would include costs related to the increased CT imaging of soft tissue infection, as well as costs related to the more advanced infections, requiring more aggressive management, including possible hospital admission and/or surgical intervention.

In conclusion, CT imaging referrals for assessment of upper and lower extremity soft tissue infection in the ED setting has increased during the 2-year period corresponding to the COVID-19 pandemic. There has also been an increase in various disease severity markers on CT imaging. These findings are likely multi-factorial in aetiology, and may relate to changes in social behaviour and the accessibility of healthcare resources as a result of the COVID-19 pandemic. This study highlights an important side effect of the pandemic, adding to existing literature that the impact related to COVID-19 is not limited to conditions directly caused by the virus. The future planning of healthcare systems’ response to future pandemics can be informed by further research in this field including targeted strategies to prevent, diagnose, and treat soft tissue infection early in its course, in an effort to mitigate delayed presentation of more advanced disease to the ED.

## Supplementary Material

tzae016_Supplementary_Data

## References

[1] COVID-19 Public Health Emergency of International Concern (PHEIC) Global research and innovation forum. Accessed March 13, 2024. https://www.who.int/publications/m/item/covid-19-public-health-emergency-of-international-concern-(pheic)-global-research-and-innovation-forum.

[2] Cucinotta D , Vanelli M. WHO declares COVID-19 a pandemic. Acta Biomed. 2020;91(1):157-160. 10.23750/ABM.V91I1.9397 32191675 PMC7569573

[3] Murti M , Whelan M, Saunders A, Hohenadel K, Gubbay J, Buchan S. Nosocomial infection surveillance: surveillance of persons-who tested negative for COVID-19 in Ontario, January 22–February 22, 2020. Can Commun Dis Rep. 2020;46(5):150-154. 10.14745/CCDR.V46I05A08 32558821 PMC7279126

[4] Fayad LM , Carrino JA, Fishman EK. Musculoskeletal infection: role of CT in the emergency department. Radiographics. 2007;27(6):1723-1736. 10.1148/RG.276075033/ASSET/IMAGES/LARGE/G07NV10G26X.JPEG 18025514

[5] Turecki MB , Taljanovic MS, Stubbs AY, et al Imaging of musculoskeletal soft tissue infections. Skeletal Radiol. 2010;39(10):957-971. 10.1007/S00256-009-0780-0/FIGURES/23 19714328

[6] Altmayer S , Verma N, Dicks EA, Oliveira A. Imaging musculoskeletal soft tissue infections. Semin Ultrasound CT MR. 2020;41(1):85-98. 10.1053/J.SULT.2019.09.005 31964497

[7] Kwee RM , Kwee TC. Diagnostic performance of MRI and CT in diagnosing necrotizing soft tissue infection: a systematic review. Skeletal Radiol. 2021;51(4):727-736. 10.1007/S00256-021-03875-9 34302500

[8] Pineda C , Espinosa R, Pena A. Radiographic imaging in osteomyelitis: the role of plain radiography, computed tomography, ultrasonography, magnetic resonance imaging, and scintigraphy. Semin Plast Surg. 2009;23(2):80-89. 10.1055/S-0029-1214160 20567730 PMC2884903

[9] COVID-19: hospitalizations & deaths—City of Toronto. Accessed March 13, 2024. https://www.toronto.ca/home/covid-19/covid-19-pandemic-data/covid-19-hospitalizations-deaths/

[10] McGee SA , Barnum M, Nesbit RD. The epidemiology of necrotizing fasciitis at a rural level 1 trauma center during the COVID-19 pandemic. Am Surg. 2022;89(5):1713-1718. 10.1177/00031348221074251 35128968 PMC8819579

[11] Nguyen QD , Diab J, Khaicy D, et al The impact of COVID-19 on delayed presentations of necrotising fasciitis. J Surg Case Rep. 2022;2022(2):rjac015. 10.1093/JSCR/RJAC015 35145627 PMC8826021

[12] McRae M , Sardiwalla Y, Nachmani O, Price E, Huynh M, Coroneos C. Upper extremity infection related to intravenous drug use: considering the true cost of the COVID-19 pandemic and lockdown. Hand (N Y). 2022;18(6):999-1004. 10.1177/15589447221077377 35193427 PMC10470235

[13] Glazier RH , Green ME, Wu FC, Frymire E, Kopp A, Kiran T. Shifts in office and virtual primary care during the early COVID-19 pandemic in Ontario, Canada. CMAJ. 2021;193(6):E200-E210. 10.1503/CMAJ.202303/TAB-RELATED-CONTENT 33558406 PMC7954541

[14] Canadian Institute of Health Information. COVID-19’s impact on physician services. 2020. Accessed March 13, 2024. https://www.cihi.ca/en/covid-19-resources/impact-of-covid-19-on-canadas-health-care-systems/physician-services

[15] ICES. The Impact of COVID-19 on Ontario’s Health Care System. 2021. Accessed March 13, 2024. https://www.ices.on.ca/Newsroom/Impact-Stories/2020-21/The-impact-of-COVID-19-on-Ontarios-health-care-system.

[16] Knight BD , Shurgold J, Smith G, et al The impact of COVID-19 on community antibiotic use in Canada: an ecological study. Clin Microbiol Infect. 2022;28(3):426-432. 10.1016/J.CMI.2021.10.013 34757115 PMC8556063

[17] Maringe C , Spicer J, Morris M, et al The impact of the COVID-19 pandemic on cancer deaths due to delays in diagnosis in England, UK: a national, population-based, modelling study. Lancet Oncol. 2020;21(8):1023-1034. 10.1016/S1470-2045(20)30388-0/ATTACHMENT/52C550CA-E7E3-475D-8B04-14D4EDC4444D/MMC1.PDF 32702310 PMC7417808

[18] Majeed A , Wright T, Guo B, Arora RS, Lam CG, Martiniuk AL. The global impact of COVID-19 on childhood cancer outcomes and care delivery—a systematic review. Front Oncol. 2022;12:869752. 10.3389/FONC.2022.869752 35463381 PMC9023072

[19] Riera R , Bagattini ÂM, Pacheco RL, Pachito DV, Roitberg F, Ilbawi A. Delays and disruptions in cancer health care due to COVID-19 pandemic: systematic review. JCO Glob Oncol. 2021;7(7):311-323. 10.1200/GO.20.00639 33617304 PMC8081532

[20] Oropallo A. COVID-19: Issues related to wound care and telehealth management. 2022. Accessed March 13, 2024. https://www.uptodate.com/contents/covid-19-issues-related-to-wound-care-and-telehealth-management

[21] Ramani SL , Samet J, Franz CK, et al Musculoskeletal involvement of COVID-19: review of imaging. Skeletal Radiol. 2021;50(9):1763-1773. 10.1007/S00256-021-03734-7 33598718 PMC7889306

[22] Gehlhausen JR , Little AJ, Ko CJ, et al Lack of association between pandemic chilblains and SARS-CoV-2 infection. Proc Natl Acad Sci U S A. 2022;119(9). 10.1073/PNAS.2122090119 PMC8892496 35217624

[23] Hayeri MR , Ziai P, Shehata ML, Teytelboym OM, Huang BK. Soft-tissue infections and their imaging mimics: from cellulitis to necrotizing fasciitis. Radiographics. 2016;36(6):1888-1910. 10.1148/RG.2016160068 27726741

[24] Lee AH , Berlyand Y, Dutta S, et al CT utilization in evaluation of skin and soft tissue extremity infections in the ED: Retrospective cohort study. Am J Emerg Med. 2023;64:96-100. 10.1016/J.AJEM.2022.11.035 36502653

