# Supplementary material for: Emergency department referrals for CT imaging of extremity soft tissue infection: before and during the COVID-19 pandemic
Source: BJR Open. 2024 Aug 22;6(1):tzae016. doi: 10.1093/bjro/tzae016 (PMC11399226; doi:10.1093/bjro/tzae016)
Supplement: tzae016_Supplementary_Data [file tzae016_supplementary_data.docx]

**Supplementary Table:** Comparison of anatomical regions involved in soft tissue infection in patients presenting before and during the COVID-19 pandemic

|  | | **Pre-COVID cohort**  **(Feb 2018 – Jan 2020** | **COVID cohort**  **(Feb 2020 – Jan 2022)** |
| --- | --- | --- | --- |
| **Anatomical region involved on CT** | Shoulder-Arm  [n] (% of positive scans) | 6 (2.93) | 15 (4.1) |
|  | Elbow-Forearm  [n] (% of positive scans) | 19 (9.3) | 22 (6.0) |
|  | Wrist-Hand  [n] (% of positive scans) | 10 (4.9) | 20 (5.5) |
|  | Shoulder-Forearm  [n] (% of positive scans) | 1 (0.5) | 6 (1.6) |
|  | Elbow-Hand  [n] (% of positive scans) | 11 (5..4) | 12 (3.3) |
|  | Shoulder-Hand  [n] (% of positive scans) | 2 (1.0) | 3 (0.8) |
|  | Hip-Thigh  [n] (% of positive scans) | 31 (15.1) | 58 (15.8) |
|  | Knee-Calf  [n] (% of positive scans) | 14 (6.8) | 20 (5.5) |
|  | Ankle-Foot  [n] (% of positive scans) | 62 (30.2) | 119 (32.4) |
|  | Hip-Calf  [n] (% of positive scans) | 1 (0.5) | 4 (1.1) |
|  | Knee-Foot  [n] (% of positive scans) | 41 (20) | 73 (19.9) |
|  | Hip-Foot  [n] (% of positive scans) | 7 (3.4) | 15 (4.1) |
